# Lectin receptor kinases in plant innate immunity

**DOI:** 10.3389/fpls.2013.00124

**Published:** 2013-05-07

**Authors:** Prashant Singh, Laurent Zimmerli

**Affiliations:** ^1^Department of Life Science, National Taiwan UniversityTaipei, Taiwan; ^2^Institute of Plant Biology, National Taiwan UniversityTaipei, Taiwan

**Keywords:** plant, receptor-like kinase, lectin receptor kinase, innate immunity, stomatal innate immunity, bacteria

## Abstract

A key feature of innate immunity is the ability to recognize and respond to potential pathogens in a highly sensitive and specific manner. In plants, the first layer of defense is induced after recognition by pattern recognition receptors of microbe-associated molecular patterns. This recognition elicits a defense program known as pattern-triggered immunity. Pathogen entry into host tissue is a critical early step in causing infection. For foliar bacterial pathogens, natural surface openings such as stomata, are important entry sites. Stomata in contact with bacteria rapidly close and can thus restrict bacterial entry into leaves. The molecular mechanisms regulating stomatal closure upon pathogen perception are not yet well-understood. Plant lectin receptor kinases are thought to play crucial roles during development and in the adaptive response to various stresses. Although the function of most plant lectin receptor kinases is still not clear, a role for this kinase family in plant innate immunity is emerging. Here, we summarize recent progresses in the identification of lectin receptor kinases involved in plant innate immunity. We also discuss the role of lectin receptor kinases in stomatal innate immunity signaling.

## INTRODUCTION

Plants face threats from various pathogenic microbes and resist attacking pathogens through both constitutive and inducible defenses (Jones and Dangl, 2006). The pattern-triggered immunity (PTI) defense response represents the front line of plant innate immunity. PTI is activated upon recognition of pathogen- or microbe-associated molecular patterns (PAMPs or MAMPs) via pattern recognition receptors (PRRs; Jones and Dangl, 2006; Zipfel, 2009; Tsuda and Katagiri, 2010; Zhang and Zhou, 2010). Examples of MAMPs comprise the lipopolysaccharide envelope of Gram-negative bacteria, peptidoglycans from Gram-positive bacteria, eubacterial flagellin, eubacterial elongation factor (EF), methylated bacterial DNA fragments, and fungal cell wall derived glucans, chitins, and proteins (Girardin et al., 2002; Cook et al., 2004; Ausubel, 2005; Boller and Felix, 2009). MAMP perception results in PTI activation which includes downstream defense responses such as production of reactive oxygen species (ROS), activation of mitogen-activated protein kinases, changes in gene expression, and production of defense compounds together leading to broad resistance to pathogens (Boller and Felix, 2009). In addition, MAMP perception at stomatal guard cells induces stomatal closure, thus activating stomatal innate immunity (Melotto et al., 2006; Zeng et al., 2010).

Pathogen entry into host tissue is a critical, first step in causing plant infection. Stomata at the leaf epidermis are natural openings that bacteria use to enter into leaves. Typically, *Arabidopsis* stomata close when in contact with bacteria, thus functioning as innate immunity gates to actively prevent bacteria entry into plants (Melotto et al., 2006, 2008, Schulze-Lefert and Robatzek, 2006; Zeng et al., 2010; Faulkner and Robatzek, 2012). Usually, 1 h after exposure to *Pseudomonas syringae* pv. *tomato* strain DC3000 (*Pst* DC3000) bacteria, *Arabidopsis* stomata close as a result of stomatal innate immunity activation. Virulent bacteria such as *Pst* DC3000 can re-open *Arabidopsis* Col-0 stomata 3–4 h after infection through the action of the chemical effector coronatine (COR) suggesting that plant pathogens have evolved virulence factors to suppress innate immunity functions of stomata (Melotto et al., 2006; Schulze-Lefert and Robatzek, 2006). The ability of COR to inhibit stomatal closure is dependent on the *COI1* gene (Melotto et al., 2006) and the priming compound beta-aminobutyric acid (BABA) blocks the COR-dependent re-opening of stomata during *Pst* DC3000 and *Pectobacterium carotovorum* ssp*. carotovorum* (*Pcc*) infection (Tsai et al., 2011; Po-Wen et al., 2013). Stomatal closure in response to treatments with flg22, a peptide representing the most conserved domain of bacterial flagellin, is dependent on the flagellin receptor FLS2 (FLAGELLIN SENSITIVE2), demonstrating that perception of bacterial MAMPs through PRRs leads to closure of *Arabidopsis* stomata (Zipfel et al., 2004; Zeng and He, 2010). The chloroplastic enzyme ASPARTATE OXIDASE that catalyzes *de novo* biosynthesis of nicotinamide adenine dinucleotide is also a critical player during activation of stomatal innate immunity in response to *Pst* infection (Macho et al., 2012). In addition, both salicylic acid (SA) and abscisic acid (ABA) signaling pathways are required during bacteria- and MAMP-induced stomatal closure in *Arabidopsis* (Melotto et al., 2006; Zeng et al., 2010). Recent works emphasized the lectin receptor kinases in plant innate immunity. In this review, we will thus focus on the role of this emerging family of receptor kinases in plant innate immunity, with highlights on stomatal innate immunity.

## LECTIN RECEPTOR KINASES IN PLANT DEFENSE

In plants, perception and transduction of environmental stimuli are largely governed by receptor-like kinases (RLKs; Mahajan and Tuteja, 2005). RLKs belong to a vast protein family found in higher plants that is represented by 610 genes in the *Arabidopsis* genome (Shiu and Bleecker, 2001, 2003).Lectin receptor kinases are RLKs characterized by an extracellular lectin motif. These lectin receptor kinases are classified into three types: G, C, and L (Bouwmeester and Govers, 2009; Vaid et al., 2012). G-type lectin receptor kinases are known as S-domain RLKs and are involved in self-incompatibility in flowering plants (Kusaba et al., 2001; Sherman-Broyles et al., 2007). C-type (calcium-dependent) lectin motifs can be found in a large number of mammalian proteins that mediate innate immune responses and play a major role in pathogen recognition (Cambi et al., 2005), but are rare in plants. *Arabidopsis* has only a single gene encoding a protein with a C-type lectin motif but so far its function has not been elucidated (Bouwmeester and Govers, 2009). *Arabidopsis* contains 45 L-type lectin receptor kinases (LecRKs) that are characterized by an extracellular legume lectin-like domain, a transmembrane domain and an intracellular kinase domain (Herve et al., 1996; Barre et al., 2002; Bouwmeester and Govers, 2009). LecRKs were suggested to play a role in abiotic stress signal transduction (Garcia-Hernandez et al., 2002; Nishiguchi et al., 2002; Riou et al., 2002; He et al., 2004; Deng et al., 2009; Joshi et al., 2010). Notably, LecRK members of the *Arabidopsis* LecRK-VI clade (Bouwmeester and Govers, 2009), are redundant negative regulators of the ABA response during seed germination (Xin et al., 2009).

Due to the resemblance of the extracellular domain with lectin proteins known to bind to fungal and bacterial cell wall components, lectin receptor kinases are predominantly hypothesized to participate in biotic stress tolerance (Bouwmeester and Govers, 2009). Some lectin receptor kinases were indeed reported to be involved in plant resistance to pathogens. For example, *Pi-d2*, a G-type lectin receptor kinase from rice, provides resistance against the fungal pathogen *Magnaporthe grisea*, the causal agent of rice blast (Chen et al., 2006). In tobacco, the expression of another G-type lectin receptor kinase was recently shown to be up-regulated by lipopolysaccharides (Sanabria et al., 2012). In *Nicotiana benthamiana,* the LecRK *NbLRK1* was suggested to be a component of the *N. benthamiana* protein complex that recognizes the *Phytophthora infestans* INF1 elicitor and mediates INF1-induced cell death (Kanzaki et al., 2008).

Like few other RLK proteins, such as PERK (proline-rich extensin-like receptor protein kinase), WAK (wall-associated kinase) and CrRLK (*Catharanthus roseus-*like RLK), LecRK-I.9 mediates cell wall–plasma membrane (CW–PM) continuum (Bouwmeester and Govers, 2009). The maintenance of structural CW–PM continuity is a critical factor that governs plants response to various stimuli and is essential for defense against pathogens (Bouwmeester and Govers, 2009; Bouwmeester et al., 2011). The association of RGD (arginine–glycine–aspartic acid) motif containing proteins with cellular proteins is a key mechanism that maintains the structural integrity of CW–PM contacts (Gouget et al., 2006). The RGD motif present in IPI-O (*in planta* induced-O), a secreted effector protein of the oomycete pathogen *Phytophthora infestans*, disrupts CW–PM adhesions upon interaction with a variety of cellular proteins, including LecRKs (Gouget et al., 2006). Further analysis revealed that deficiency in LecRK-I.9, earlier found to interact with RGD motif containing proteins (Gouget et al., 2006), leads to a gain of susceptibility phenotype toward the oomycete *Phytophthora brassicae* (Bouwmeester et al., 2011). These results imply that LecRKs may be involved in protein–protein interactions with RGD-containing proteins as potential ligands, and may play a structural and signaling role at the plant cell surfaces upon pathogen infection.

LecRK-VI.2 is critical for resistance against hemibiotrophic *Pst* DC3000 and necrotrophic *Pcc* bacteria (Singh et al., 2012). Increased susceptibility of the transferred DNA (T-DNA) insertion mutant line *lecrk-VI.2-1* is correlated with defective bacteria- and MAMP-induced MPK3 (Mitogen-activated protein kinase 3) and MPK6 (Mitogen-activated protein kinase 6) activities, PTI-responsive gene expression, and callose deposition (Singh et al., 2012). Transcriptome analysis of a *LecRK-VI.2* over-expression line revealed transcription up-regulation of numerous genes responsive to virulent or avirulent bacteria, the MAMP flg22, or to the SA functional analog benzothiadiazole further suggesting a role for LecRK-VI.2 in the *Arabidopsis* PTI response (Singh et al., 2012). BAK1 (Brassinosteroid insensitive1-associated kinase 1) and FLS2 association, BIK1 (BOTRYTIS-INDUCED KINASE1) phosphorylation, and ROS production that are usually considered as early PTI responses (Zipfel and Robatzek, 2010), were not compromised in the mutant *lecrk-VI.2-1*. These data suggest that LecRK-VI.2 positively modulates PTI signaling upstream of MPK3 and MPK6 and downstream of FLS2 (Singh et al., 2012). In addition, LecRK-VI.2 is a key modulator of BABA-mediated priming and BABA-induced resistance (Singh et al., 2012). Further analyses of the function of LecRK-VI.2 revealed that LecRK-VI.2 possesses a functional kinase domain and is not critical for resistance to the necrotrophic fungal pathogen *Botrytis cinerea* (Singh et al., 2013). By contrast, over-expression of the plasma membrane-localized L-type lectin-like protein kinase 1, AtLPK1 (LecRK-IV.3) induces *Arabidopsis* resistance to *B. cinerea* (Huang et al., 2013).

Lectin receptor kinases are also critical for plant resistance to insects. The lectin receptor kinase 1 (LecRK1) is important during herbivory by *Manduca sexta* larvae to suppress insect-mediated inhibition of jasmonic acid-induced defense responses in *Nicotiana attenuata* (Gilardoni et al., 2011). Importantly, reduction of *LecRK1* expression in *N. attenuata* induces increased *Manduca sexta* folivory (Gilardoni et al., 2011). The insect-induced accumulation of protease inhibitors, as well as the expression of the gene encoding threonine deaminase, two critical defense responses were also several fold reduced in *N. attenuata* with a silenced *LecRK1* when compared to non-silenced controls (Gilardoni et al., 2011). Inhibition of SA accumulation through the expression of nahG in silenced *lecRK1* plants restores wild-type levels of resistance against *Manduca sexta* herbivory, suggesting that LecRK1 inhibits the accumulation of SA during herbivory (Gilardoni et al., 2011). More recently, LecRK-I.8 was suggested to be important for the perception of insect egg-derived elicitors in *Arabidopsis* (Gouhier-Darimont et al., 2013).

## LecRK-VI.2 AND LecRK-V.5 IN *Arabidopsis* STOMATAL INNATE IMMUNITY

In addition to positively regulating apoplastic PTI, LecRK-VI.2 is also critical for *Arabidopsis* stomatal innate immunity (Singh et al., 2012). Notably and similarly to the PRR mutant *fls2* (Zeng and He, 2010), *lecrk-VI.2-1* mutants demonstrate a high sensitivity to *Pst* DC3000 COR^-^ deficient bacterial mutants that cannot re-open stomata upon infection. Since *Arabidopsis* is resistant to these bacterial mutants (Melotto et al., 2006), LecRK-VI.2 may play a positive role in bacteria-mediated stomatal closure (Singh et al., 2012). Consistent with this observation, stomatal closure upon bacterial inoculation and MAMPs treatments were found to be defective in the mutant *lecrk-VI.2-1* (Singh et al., 2012). This suggests that LecRK-VI.2 plays a positive role during stomatal innate immunity activation at a signaling node downstream of MAMP perception. In addition, transgenic lines over-expressing *LecRK-VI.2* demonstrate constitutive stomatal closure, further suggesting a positive role for LecRK-VI.2 in stomatal innate immunity (Singh et al., 2012). The mutant *lecrk-VI.2-1* demonstrates wild-type stomatal closure levels in response to ABA indicating that LecRK-VI.2 acts upstream or independently of ABA signaling during stomatal closure (Singh et al., 2012).

Another LecRK involved in *Arabidopsis* stomatal innate immunity is LecRK-V.5. However, in contrary to LecRK-VI.2 that positively regulates stomatal innate immunity, LecRK-V.5 negatively regulates stomatal closure upon bacterial infection. Plants lacking a functional LecRK-V.5 are resistant to *Pst* DC3000 and *Pcc* surface inoculation, but are normally sensitive to infiltration inoculation (Arnaud et al., 2012; Desclos-Theveniau et al., 2012). These observations suggest that disruption of LecRK-V.5 affects early *Arabidopsis* defenses by restricting bacterial entry into leaves and point to a role of LecRK-V.5 in stomatal innate immunity (Desclos-Theveniau et al., 2012). Analyses of stomatal apertures in *lecrk-V.5* indeed revealed that this mutant possesses constitutively closed stomata (Desclos-Theveniau et al., 2012). Transgenic lines over-expressing *LecRK-V.5* are less resistant to *Pst* DC3000 COR^-^ and this is correlated with a re-opening of stomata in *LecRK-V.5* over-expression lines even in the absence of COR. These observations suggest the existence of a stomatal re-opening mechanism positively modulated by LecRK-V.5 (Desclos-Theveniau et al., 2012). Interestingly, LecRK-V.5 over-expression lines are also defective in MAMP-induced stomatal closure. Together these data indicate that LecRK-V.5 negatively regulates *Arabidopsis* resistance to bacteria through fine-tuning of stomatal innate immunity (Desclos-Theveniau et al., 2012). Localized expression of LecRK-V.5 upon PTI activation at stomatal guard cells further supports a role for LecRK-V.5 in stomatal innate immunity (Desclos-Theveniau et al., 2012). Similarly to the *scord5* mutant that shows a defective stomatal innate immunity but exhibits wild-type apoplastic immunity (Zeng et al., 2011), apoplastic PTI responses such as flg22-triggered oxidative burst, bacteria-mediated callose deposition and up-regulation of PTI marker genes are not affected in *lecrk-V.5* mutants. COR treatments re-open closed stomata in *lecrk-V.5* mutants (Desclos-Theveniau et al., 2012), suggesting that LecRK-V.5 acts upstream of COR. *lecrk-V.5* mutants accumulate high levels of ROS in guard cells and chemical inhibition of ROS accumulation in *lecrk-V.5* guard cells re-opens closed stomata (Desclos-Theveniau et al., 2012). By contrast, treatments with PAMPs increase guard cell ROS levels in wild-type, but no increase of ROS production was observed in *Arabidopsis* over-expressing *LecRK-V.5* (Desclos-Theveniau et al., 2012). Since ROS induce stomatal closure, high levels of ROS, and defective ROS accumulation may explain constitutive stomatal closure in *lecrk-V.5* mutants and deficient stomatal closure in *LecRK-V.5* over-expression lines, respectively. In addition, lines over-expressing *LecRK-V.5* demonstrate a compromised ABA-mediated stomatal closure (Desclos-Theveniau et al., 2012), thus LecRK-V.5 functions in guard cell ABA signaling pathway downstream of MAMP perception. LecRK-V.5 may thus act at a specific branch involving ABA for the control of stomatal innate immunity and may negatively regulate ABA-mediated stomatal responses (Desclos-Theveniau et al., 2012). Negative regulation of stomatal innate immunity may have evolved in order to avoid the deleterious effects of a prolonged inhibition of photosynthesis that would be caused by decreased CO_2_ availability following prolonged stomatal closure.

## CONCLUSION

Although new knowledge about lectin receptor kinases function and signaling has emerged recently, many questions still remain unanswered. For example, what are the potential ligands and downstream partners that modulate lectin receptor kinase-dependent innate immunity responses are critical points that need to be solved. Importantly, the unraveling of the mechanisms modulating ligands perception by lectin receptor kinases will provide further insights into how LecRKs affect the plant response to pathogens. This may clarify whether these receptor kinases function as PRRs. Knowledge derived from such studies could lead to novel methods for managing plant disease resistance.

## Conflict of Interest Statement

The authors declare that the research was conducted in the absence of any commercial or financial relationships that could be construed as a potential conflict of interest.
